#  The role of serum and bronchoalveolar lavage fluid chitotriosidase activity on diagnosis, disease characteristics and prognosis of sarcoidosis

**DOI:** 10.5578/tt.20239605

**Published:** 2023-12-06

**Authors:** Gözde KÖYCÜ BUHARİ, Aydın ÇİLEDAČ, İsmail KURT, Emel ÇAČLAR, Akın KAYA, Özlem ÖZDEMİR KUMBASAR, Gökhan ÇELİK

**Affiliations:** 1 Department of Immunology and Allergy, Ankara Atatürk Sanatoryum Training and Research Hospital, University of Health Sciences, Ankara, Türkiye; 2 Department of Pulmonary Diseases, Ankara University Faculty of Medicine, Ankara, Türkiye; 3 Department of Biochemistry (Retired Lecturer), Gülhane Training and Research Hospital, University of Health Sciences, Ankara, Türkiye; 4 Department of Biochemistry, Gülhane Training and Research Hospital, University of Health Sciences, Ankara, Türkiye

## Abstract

**ABSTRACT**

** The role of serum and bronchoalveolar lavage fluid chitotriosidase activity on diagnosis, disease characteristics and prognosis of sarcoidosis **

**Introduction:** *Sarcoidosis is a multisystem granulomatous disease with an unpredictable clinical course. Chitotriosidase is a chitinase mainly expressed by activated macrophages. Increased chitotriosidase activity has been report- ed in serum and bronchoalveolar lavage (BAL) of sarcoidosis patients com- pared to healthy controls. This study aims to evaluate the role of serum and BAL chitotriosidase activity on diagnosis, disease characteristics, and progno- sis of sarcoidosis.
*

**Materials and Methods:** *Patients referred with suspected sarcoidosis or other interstitial lung disease were prospectively included in the study. All patients underwent bronchoscopy with BAL. Serum and BAL chitotriosidase activity, BAL differential cell counts, and lymphocyte phenotypes were determined. Sarcoidosis patients were followed up regularly. *

**Results:** *Forty-two sarcoidosis and 28 non-sarcoidosis patients were included in the study. Serum chitotriosidase activity was higher in sarcoidosis group *

* 247.5 (2.78-461) vs 108 (2.78-272) nmol/h/mL (p< 0.001). BAL chitotriosi- dase activity tended to be higher in sarcoidosis group 11 (2-308) vs 6.95 (2.27-44) nmol/h/mg but was not found to be statistically significant (p= 0.11). Serum and BAL chitotriosidase activities were correlated with each other (p= 0.023, r= 0.355). No significant difference was found between the diagnostic performance of BAL CD4/CD8 ratio and serum chitotriosidase activity (p= 0.079). Serum chitotriosidase and ACE activities were correlated with each other (p= 0.004, r= 0.457). No significant difference was found *

* between serum or BAL chitotriosidase activity and stage or extrapulmonary involvement. Serum chitotriosidase activity was higher in patients who needed systemic therapy at diagnosis (p= 0.046). However, no significant difference was found between serum or BAL chitotriosidase activities and disease progression (p= 0.395 and p= 0.723, respectively). *

**Conclusion:** *Serum chitotriosidase activity can be helpful in the differential diagnosis of sarcoidosis with a similar diagnostic perfor- mance with BAL CD4/CD8 ratio. Although serum chitotriosidase activity at diagnosis does not predict progressive disease, it is associated with the need for systemic therapy at diagnosis. Serial chitotriosidase measurements may be useful in monitoring disease progression during follow-up. *

**Key words:** *Chitotriosidase; sarcoidosis; bronchooalveolar lavage; interstitial lung disease *

**ÖZ**

** Serum ve bronkoalveolar lavaj sıvısı chitotriosidase aktivitesinin sarkoidoz tanısı, hastalık özellikleri ve prognozundaki rolü **

**Giriş:** *Sarkoidoz, klinik seyri öngörülemeyen multisistem granülomatöz bir hastalıktır. Chitotriosidase, esas olarak aktifleştirilmiş mak- rofajlar tarafından eksprese edilen bir kitinazdır. Sarkoidoz hastalarında sağlıklı kontrollerle karşılaştırıldığında serum ve bronkoalveo- lar lavajda (BAL) artmış chitotriosidase aktivitesi bildirilmiştir. Bu çalışmanın amacı serum ve BAL chitotriosidase aktivitesinin sarkoi- doz tanısı, hastalık özellikleri ve prognozundaki rolünü değerlendirmektir.
*

**Materyal ve Metod:** *Sarkoidoz veya diğer interstisyel akciğer hastalıkları şüphesi ile sevk edilen hastalar prospektif olarak çalışmaya dahil edildi. Tüm hastalara bronkoskopi ve BAL yapıldı. Serum ve BAL chitotriosidase aktivitesi, BAL diferansiyel hücre sayıları ve lenfosit fenotipleri belirlendi. Sarkoidoz hastaları düzenli olarak takip edildi. *

**Bulgular:** *Çalışmaya sarkoidoz tanısı olan 42 hasta ve sarkoidoz tanısı olmayan 28 hasta dahil edildi. Sarkoidoz grubunda serum chitotriosidase aktivitesi daha yüksekti [247,5 (2,78-461) ve 108 (2,78-272) nmol/h/mL] (p< 0,001). BAL chitotriosidase aktivitesi sarkoidoz grubunda daha yüksek olma eğilimindeydi [6,95 (2.27-44) nmol/h/mg vs 11 (2-308)] ancak istatistiksel olarak anlamlı bulunmadı (p= 0,11). Serum ve BAL chitotriosidase aktiviteleri birbiriyle koreleydi (p= 0,023, r= 0,355). BAL CD4/CD8 oranının tanısal performansı ile serum chitotriosidase aktivitesi arasında anlamlı fark bulunmadı (p= 0,079). Serum chitotriosidase ve ACE aktiviteleri birbiriyle korele idi (p= 0,004, r= 0,457). Serum veya BAL chitotriosidase aktivitesi ile evre veya akciğer dışı tutulum ara- sında anlamlı bir fark bulunmadı. Tanı anında sistemik tedavi ihtiyacı olan hastalarda serum chitotriosidase aktivitesi daha yüksekti (p= 0,046), ancak serum veya BAL chitotriosidase aktiviteleri ile hastalık progresyonu arasında anlamlı fark bulunmadı (sırasıyla p= 0,395 ve p= 0,723). *

**Sonuç:** *Serum chitotriosidase aktivitesi, BAL CD4/CD8 oranı ile benzer bir tanı performansı ile sarkoidozun ayırıcı tanısında yardımcı olabilir. Tanı anındaki serum chitotriosidase aktivitesi ilerleyici hastalığı öngörmese de tanı anında sistemik tedavi ihtiyacı ile ilişkilidir. Seri chitotriosidase ölçümleri, takipte hastalık ilerlemesinin izlenmesinde faydalı olabilir. *

**Anahtar kelimeler:** *Chitotriosidase; sarkoidoz; bronkoalveolar lavaj; interstisyel akciğer hastalığı *

## INTRODUCTION

Sarcoidosis is a multisystem non-caseating granulomatous disease of unknown origin characterized by T-lymphocyte activation and accumulation of CD4-positive T-lymphocytes in the organs involved, most commonly the lungs (1,2). There is no single diagnostic test that helps confirm the disease. In clinical practice, a diagnosis of sarcoidosis is established upon the presence of a compatible clinical and/or radiological profile, alongside histopathologically confirmed non- caseating granulomas. This diagnosis inherently involves the exclusion of other diseases exhibiting similar clinical or histopathological characteristics (1,3). The clinical course of sarcoidosis is unpredictable. Generally, it has a good prognosis and spontaneous remission may occur. However, some patients may develop progressive interstitial disease leading to end-stage fibrosis (4). The unpredictable

clinical course of sarcoidosis has prompted research into biomarkers that could effectively predict disease activity and outcomes. Chitotriosidase (CTO) stands out as one of the promising biomarkers for both diagnosing and prognosticating sarcoidosis.

CTO is an enzyme that belongs to the chitinase protein family. The members of this family can catalyze the hydrolysis of chitin or chitin-like substrates such as 4-methyllumbelliferyl chitotrioside (5-8). Although the role of CTO in humans is still not completely elucidated it is most likely a part of the innate immune system and involved in defense against chitin-containing pathogens such as fungi and some parasites (5,7,9).

CTO is recognized as a marker indicating macrophage stimulation, primarily originating from chronically activated tissue macrophages. Under physiological conditions, polymorphonuclear leukocytes are also capable of secreting plasma CTO (5-9). Increased

CTO activity has been documented in serum and bronchoalveolar lavage (BAL) of sarcoidosis patients compared with healthy controls (10,11). The role of CTO in disease progression is not yet understood.

This study aims to evaluate the role of serum and BAL CTO activity on diagnosis, disease characteristics, and prognosis of sarcoidosis.

### MATERIALS and METHODS

Patients who were referred to the Department of Pulmonary Diseases at Ankara University Faculty of Medicine due to suspected sarcoidosis or other interstitial lung diseases and scheduled to undergo BAL (bronchoalveolar lavage), and who consented to participate in the study, were prospectively enrolled. The exclusion criteria included undetectable CTO activity in BAL or serum. Seventy-three patients were initially included in the study however three patients, two with sarcoidosis and one patient with hypersensitivity pneumonitis, were excluded from the study because of undetectable CTO activity. The study continued with a total of 70 patients.

None of the patients had been diagnosed with sarcoidosis or treated with systemic steroids or other immunosuppressants before the study. All patients gave their written informed consent to participate in the study and the study was approved by the ethics committee of Ankara University Faculty of Medicine (Approval number: 149-4614).

All patients underwent bronchoscopy with BAL. BAL samples were stained for acid-resistant bacillus and cultured for bacteria and mycobacteria to exclude infections. BAL differential cell counts were determined and BAL lymphocyte phenotypes were analyzed by flow cytometry. Cells were separated by centrifuge and the fluid fraction was stored at -80 °C until BAL CTO assay. Venous blood samples were collected simultaneously with bronchoscopy, centrifugated and serum samples were stored at

-80 °C until serum CTO assay.

Chitotriosidase activity was measured according to the method described previously by Guo et al (12). In summary, 5 µL of serum was incubated with 100 µL of 4-methylumbelliferyl-β-D-N,N’,N’’- triacetylchitotriose (Sigma M-5639) in McIlvain’s phosphate-citrate buffer, pH= 5.2, for one hour at 37 °C. The reaction was terminated by adding 120

µL 0.5 mol/L Na2CO3-NaHCO3 buffer at a pH of

10.7. Subsequently, the fluorescence of 4

methylumbelliferon was measured in a Microfluor 2® fluorimeter (BIO-TEK SynergyHT; excitation 355, emission 460 nm). The chitotriosidase activity was expressed as nanomoles of substrate hydrolyzed per mL per hour (nmol/mL/h). Total protein concentrations in BAL fluid were determined by Lowry method (13) and BAL CTO activity was corrected according to BAL protein concentrations. BAL CTO activity was expressed as nmol/h/mg.

During the bronchoscopy procedure, alongside bronchoalveolar lavage (BAL), transbronchial needle aspiration (TBNA) and bronchus mucosa biopsy (BMB) were also commonly conducted in most cases. In instances where it was considered necessary, mediastinoscopic lymph node sampling and extrapulmonary tissue biopsy were conducted.

All patients underwent pulmonary function tests (including FVC, FEV1, DLCO, and TLC), blood gas analysis, chest X-ray, thoracic computerized

tomography (CT), and high-resolution computerized tomography (HRCT). When deemed necessary, gallium-67 scintigraphy and positron emission tomography (PET) CT scans were also performed.

Tissue markers for systemic autoimmune diseases, including anti-nuclear antibody (ANA), anti- neutrophil cytoplasmic antibodies (ANCA), anti- double-strained DNA (anti-dsDNA), anti-extractable nuclear antigen (ENA) antibodies and rheumatoid factor (RF) were assessed in all patients. Serum angiotensin-converting enzyme (ACE) activity was also evaluated in patients suspected of having sarcoidosis.

Sarcoidosis diagnosis adhered to the international criteria outlined by the American Thoracic Society/ European Respiratory Society/World Association of Sarcoidosis and Other Granulomatous Disorders, which constituted the prevailing statement during the patients’ inclusion in the study (1). The diagnosis was established in a patient presenting compatible clinical, radiological, and laboratory findings, either when histopathologically proven non-caseating granulomatous inflammation was demonstrated, or when the patient exhibited a classic Löfgren syndrome (characterized by fever, erythema nodosum, arthralgias, and bilateral hilar lymphadenopathy), or when the BAL CD4/CD8 ratio was greater than 3.5, following the exclusion of other diseases that could generate a comparable histological and clinical presentation.

The radiological staging of sarcoidosis was defined according to Scadding: stage 0 (normal chest radiograph), stage I (bilateral hilar lymphadenopathy), stage II (bilateral hilar lymphadenopathy accompanied by parenchymal infiltration), stage III (parenchymal infiltration without hilar lymphadenopathy) and stage IV (advanced fibrotic disease) (14).

Sarcoidosis patients underwent screening for extrapulmonary involvement, with their calcium metabolism assessed via serum calcium sampling and 24-hour urinary calcium excretion.

Sarcoidosis patients received regular follow-up care. Initially, determination was made regarding the necessity of systemic therapy at the time of diagnosis. Patients were consistently monitored for a minimum of nine months and a maximum of 24 months from the time of diagnosis until the conclusion of the study. Throughout the follow-up period, patients were categorized into two groups: stable or progressive disease, based on pre-established criteria. Stable disease was defined as a disease with normal lung function tests at baseline, no worsening on follow-up, and no extrapulmonary involvement requiring systemic therapy. Progressive disease was defined as a disease with worsening of lung function tests (>10% decrease in FVC, >15% decrease in DLCO from baseline) or with extrapulmonary involvement requiring systemic therapy (15,16).

### Statistical Method

All statistical analyses were performed using the SPSS (Statistical Package of Social Sciences) for Windows

16.0 software package. In the evaluation of the data, mean and standard deviation for normally distributed data, median and interquartile range for data that did not show normal distribution, values, and percentages for ratios were determined by descriptive statistical method. In univariate analyses, Chi-square, Fisher, Student’s t-test, and Mann-Whitney U tests were used, as appropriate. Pearson correlation coefficient was used to examine the direction and strength of the relationship between the variables. All p-values lower than 0.05 were considered to be statistically significant.

## RESULTS

A total of 70 patients were included in the study; the diagnosis was sarcoidosis in 42 patients and non- sarcoidosis in 28 patients. The distribution of diagnoses is shown in Table 1. The diagnosis of

**Table:** 

**Table 1.** Differential diagnosis in study cohort	
**Diagnosis**	**n**
Sarcoidosis	42
Idiopathic pulmonary fibrosis	6
Tuberculosis	5
Hypersensitivity pneumonitis	4
Rheumatoid arthritis pulmonary involvement	3
Sjogren syndrome pulmonary involvement	3
Eosinophilic pneumonia	2
Cryptogenic organizing pneumonia	2
Vasculitis	2
Non-specific interstitial pneumonia	1

sarcoidosis was confirmed by histological demonstration of non-caseating granulomatous inflammation in 27 (64.3%) patients. In the remaining

15 patients it was diagnosed by the presence of clinical, radiological, and laboratory parameters consistent with sarcoidosis (e.g., Löfgren’s syndrome, asymptomatic bilateral hilar lymphadenopathy with a history of uveitis), after exclusion of malignancy and infections.

The sarcoidosis group exhibited a younger age compared to the non-sarcoidosis group, with a mean age (range) of 41.07 ± 12.83 (20-61) versus 56.39 ±

12.02 (35-78) respectively (p< 0.001). Additionally, there was a higher proportion of females in the sarcoidosis group, with a female-to-male ratio of 31/11 versus 11/17 in the non-sarcoidosis group (p= 0.004).

Although median values for FVC%, FEV1%, FEV1/ FVC and DLCO% were within normal range in the sarcoidosis group, FVC was <80% in five (11.9%) patients, FEV1 was <80% in five (11.9%) patients, FEV1/FVC was <70 in two (4.8%) patients and DLCO was <80% in three (7.1%) patients. FVC%, FEV1%, DLCO%, and PaO2 and SaO2 levels were found to be statistically significantly higher in the sarcoidosis group than in the non-sarcoidosis group. Pulmonary function test and arterial blood gas analysis results are shown in Table 2.

Serum CTO activity was significantly higher in the sarcoidosis group than in the non-sarcoidosis group [247.5 (2.78-461) vs 108 (2.78-272) nmol/h/mL,

respectively (p< 0.001)]. BAL CTO activity tended to be higher in the sarcoidosis group than the non- sarcoidosis group but was not statistically significant

**Table:** 

**Table 2.** Pulmonary function test and arterial blood gas analysis results of sarcoidosis and non-sarcoidosis patients			
	**Sarcoidosis patients,** **median (min-max)**	**Non-sarcoidosis patients,** **median (min-max)**	**p**
PaO2, mmHg	76 (46-92)	67 (41-82)	0.001
PaCO2, mmHg	34 (21-55)	34 (23-39)	0.850
pH	7.43 (7.37-7.52)	7.43 (7.36-7.47)	0.674
SaO2%	95 (84-99)	94 (74-96)	0.009
FVC%	101 (66-142)	74 (47-104)	<0.001
FEV1%	95 (51-115)	75 (53-122)	0.007
FEV1/FVC	81 (66-92)	85 (49-93)	0.236
FEF25-75%	77 (22-149)	75 (38-93)	0.960
DLCO%	92 (55-151)	73 (36-105)	0.001
DLCO/VA	98 (57-124)	94 (58-128)	0.278

**Table:** 

**Table 3.** BAL cell profile, BAL and serum CTO activity of sarcoidosis and non-sarcoidosis patients			
	**Sarcoidosis patients, median (min-max)**	** Non-sarcoidosis patients, median (min-max) **	**p**
BAL macrophage (%)	28.50 (2-72)	40.5 (0-92)	0.087
BAL neutrophil (%)	0 (0-22)	0 (0-75)	0.158
BAL lymphocyte (%)	57 (6-85)	18 (1-50)	<0.001
BAL T cell (%)	91 (67-97)	83 (8-97)	0.001
BAL CD4/CD8 ratio	6.00 (0.20-28.00)	1.60 (0.16-0.10)	<0.001
Serum CD4/CD8 ratio	1.30 (0.50-7.00)	1.30 (0.40-5.10)	0.643
BAL CTO* activity (nmol/h/mg)	11 (2-308)	6.95 (2.27-44)	0.119
Serum CTO* activity (nmol/h/mL)	247.5 (2.78-461)	108 (2.78-272)	<0.001
*CTO: Chitotriosidase.

[11 (2-308) vs 6.95 (2.27-44) nmol/h/mg, respectively (p= 0.11)] (Table 3). Serum and BAL CTO activities were correlated with each other (p= 0.023, r= 0.355).

BAL differential cell counts and BAL lymphocyte phenotypes showed that BAL lymphocyte %, BAL T cell %, and BAL CD4/CD8 ratio were statistically significantly higher in the sarcoidosis group (Table 3).

The diagnostic accuracy of various cut-off values for BAL CD4/CD8 and serum CTO activities is presented in Table 4. The diagnostic performance of both BAL CD4/CD8 ratio and serum CTO activities in diagnosing sarcoidosis was compared using ROC analysis. The results indicated no statistically significant difference between their diagnostic rates (p= 0.079), ROC curves are shown in Figures 1A and 1B.

Within the sarcoidosis group, 15 patients exhibited high serum ACE activity (>52 IU/L), while the median ACE activity was recorded at 40 (1-214) IU/L. Serum

CTO and ACE activities were correlated with each other (p= 0.004, r= 0.457). Serum ACE activity was also correlated with the percentage of serum monocytes which are precursors of macrophages (p= 0.03, r= 0.33).

Considering the radiological stages of sarcoidosis, 13 (31%) patients had stage I and 29 (69%) patients had stage II disease. Although serum and BAL CTO activities were higher in stage II patients than stage I patients, the difference was not statistically significant (p= 0.075 and p= 0.199 respectively) (Table 5).

In addition to pulmonary involvement, 23 (54.8%) patients with sarcoidosis had extrapulmonary involvement. Of these patients, five had stage I and the remaining 18 had stage II disease. More than one extrapulmonary involvement was observed in 16 patients and all had stage II disease. Extrapulmonary organ involvements were as follows; cutaneous (n= 11, 26.2%), ocular (n= 5, 11.9%), peripheral

**Table:** 

**Table 4.** Diagnostic accuracy of different cut-off values of serum CTO activity and BAL CD4/CD8 ratio		
Serum CTO* activity (mol/h/mL)	**Sensitivity (%)**	**Specificity (%)**
137.5	71.4	70.4
185	61.9	88.9
279	45.2	100
**BAL CD4/CD8 ratio**	**Sensitivity (%)**	**Specificity (%)**
2.65	80	91
3.05	76	96
3.25	73	100
Serum CTO* activity (nmol/h/mL)	247.5 (2.78-461)	108 (2.78-272)
*CTO: Chitotriosidase.		


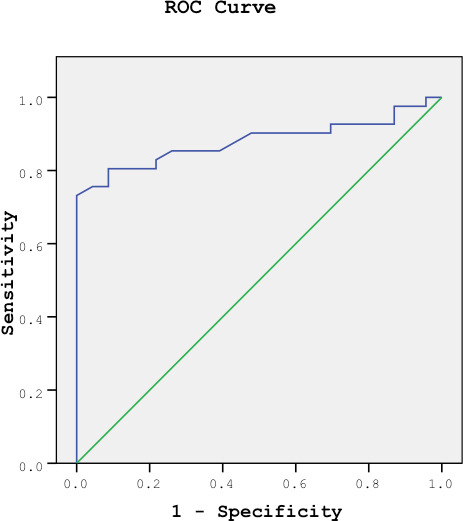
 
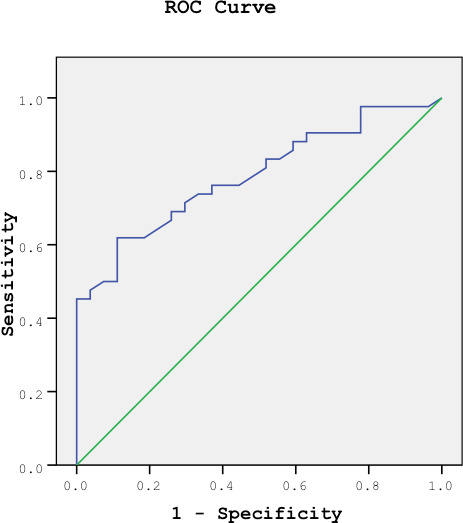

**Figure 1. (A)** ROC curve showing sensitivity and specificity of BAL CD4/CD8 ratio in sarcoidosis patients. AUC ± SD (95% CI); 0.884 ± 0.041 (0.778-0.950). **(B)** ROC curve showing sensitivity and specificity of serum CTO activity in sarcoidosis patients AUC ± SD (95% CI); 0.762 ± 0.0598 (0.638-0.860).

lymph node (n= 5, 11.9%), spleen (n= 5, 11.9%), liver (n= 3, 7.1%), parotid and lacrimal gland (n= 1, 2.4%). Calcium metabolism alterations were also observed as hypercalciuria in two patients (4.8%) and as hypercalcemia in one patient (2.4%). There were no differences between serum CTO or BAL CTO activities of cases with and without extrapulmonary involvement (p= 0.970 and p= 0.282, respectively) (Table 5). Serum CTO activity was inversely correlated with serum calcium levels (p= 0.026, r= -0.352).

Systemic therapy was initiated in five (11.90%) sarcoidosis patients at diagnosis; one for pulmonary functional abnormality, one for resistant hypercalcemia that did not respond to a calcium-

poor diet, one for lupus pernio, and two for posterior uveitis. Comparison of the patients with and without need for systemic therapy at diagnosis showed that serum CTO activity was significantly higher in patients who needed systemic therapy at diagnosis (p= 0.046), however, there was no significant difference between BAL CTO activity (p= 0.608) (Table 5).

Thirty-one of 42 sarcoidosis patients continued regular follow-up in our clinic with a minimum of nine months and a maximum of 24 months period from the time of diagnosis to the end of the study. During the follow-up period, progression was detected in three patients, prompting the initiation of systemic therapy. After excluding the five sarcoidosis

**Table:** 

**Table 5.** Clinical characteristics of sarcoidosis patients and CTO activity in serum BAL		
	Serum CTO* activity (nmol/h/mL)	BAL† CTO* activity (nmol/h/mg)
**Clinical characteristics**	**Median (min-max) p**	**Median (min-max) p**
Radiological stage		
I	139 (33.30-403.00) 0.075	5.6 (2.27-89.00) 0.199
II	289 (2.78-461)	19 (2.27-308)
Extrapulmonary involvement		
Yes	239 (2.78-461) 0.970	8.30 (2.27-219) 0.282
No	267 (34.09-408)	31 (2.27-308)
Systemic therapy at diagnosis		
Yes	386 (161-408) 0.046	25 (2.80-56) 0.608
No	229 (2.78-461)	11 (2.27-308)
Systemic therapy on follow-up		
Yes (progressive disease)	342 (239-400) 0.395	8.3(5.60-219) 0.723
No (remission or stable disease)	286 (2.78-442)	19 (2.27-308)
CTO*: Chitotriosidase, BAL†: Bronchoalveolar lavage.

patients who required systemic therapy at diagnosis and 11 patients who could not be followed up, the serum CTO and BAL CTO activities at diagnosis of the remaining 26 patients were compared to determine whether these patients showed progression or required systemic therapy during follow-up. However, no significant difference was found between the serum or BAL CTO activities (p= 0.395 and p= 0.723, respectively) (Table 5).

## DISCUSSION

In this prospective study, the diagnostic performance of serum and BAL chitotirosidase activity was evaluated in 42 sarcoidosis patients and 28 non- sarcoidosis patients. In previous studies, serum CTO activity was reported to be higher in sarcoidosis patients compared to healthy controls (11,17-21). Furthermore, serum CTO activity was reported to be in the normal range in other granulomatous lung diseases such as pulmonary tuberculosis, and other interstitial lung diseases such as idiopathic pulmonary fibrosis and pulmonary fibrosis associated with systemic sclerosis (18,19). These results suggested that this enzyme could be indicative of sarcoidosis. However, in later studies, serum CTO activity was also found to be higher than that of the controls in asbestosis, fibrosis, and lung cancer. Therefore it was suggested that CTO cannot be a specific marker of sarcoidosis (22). In our study, serum CTO activity was significantly higher in the sarcoidosis group compared to the non-sarcoidosis group. While not specific to sarcoidosis, it could still prove useful in diagnosis.

In this study, a positive correlation was shown between serum CTO and ACE activities consistent with some previous reports (11,22-24).

In this study, there was no control group consisting of healthy individuals. However, there were some reports of serum CTO activity in healthy individuals from the center where our study’s CTO enzyme activity was measured (25,26). In the first study, Kurt et al. measured serum CTO activity in 69 healthy young individuals aged between 20-44 years and in 90 healthy elderly individuals aged between 65-94 years, and found an age-dependent increase in serum CTO activity. The mean CTO activity was reported as 136 ± 17 nmol/mL/h in young individuals and 270 ±

21 nmol/mL/h in elderly individuals (26). In the second study, the reference range of CTO activity was reported as 0-90 nmol/hour/mL in 100 healthy individuals aged between 20-30 years (25). In our study, sarcoidosis patients were aged between 20-61 years old, and 83.3% (n= 35), 69.04% (n= 29) and 59.5% (n= 25) of these patients had serum CTO activity of over 100, 150, and 200 nmol/mL/h, respectively. In our study, serum ACE activity was found to be above the reference limit (52 IU/L) in 15 (35.7%) of the sarcoidosis patients. Thus, it is possible to state that serum CTO activity may be more sensitive than serum ACE activity in the diagnosis of sarcoidosis. There are other studies reporting CTO activity as a more sensitive marker than ACE activity in sarcoidosis (11,24).

Apart from age-dependent differences, another factor potentially limiting the value of serum CTO activity in diagnosing sarcoidosis is genetic CTO deficiency. CTO deficiency is caused by 24 base pairs (24-bp) duplication in the chitotriosidase gene. The frequency of 24-bp duplication shows significant variations among countries and continents (27). Individuals with the heterozygous genotype for 24-bp duplication have approximately half as much CTO activity as individuals with the homozygous wild genotype, while homozygous mutant individuals have no CTO activity in serum or plasma (28). Kurt et al. reported heterozygosity and homozygosity frequency of the 24-bp duplication in the Turkish population as 36% and 8% respectively (29). In our study, two patients with sarcoidosis and one patient with hypersensitivity pneumonitis were excluded from the study due to undetectable CTO activity.

BAL is a useful procedure for the differential diagnosis of interstitial lung diseases and the identification of granulomatous lung diseases. An essential goal of research is to discover new diagnostic biomarkers in the serum or BAL of sarcoidosis patients to avoid histological examination. Limited data exist regarding CTO activity in the BAL of patients with sarcoidosis and other interstitial lung diseases. BAL CTO activity was reported to be significantly higher in sarcoidosis patients than controls (10,18,24,30). Furthermore, idiopathic pulmonary fibrosis patients, but not systemic sclerosis patients were reported to have significantly higher BAL CTO activity than controls

(18). In another study, BAL CTO activity was not found to be different between other interstitial lung diseases and sarcoidosis (31). In our study, BAL CTO activity tended to be higher in the sarcoidosis group but was not statistically significant, and serum and BAL CTO activities were found to be correlated with each other in sarcoidosis patients consistent with previous data (30). Further studies are warranted to evaluate the role of BAL CTO activity in sarcoidosis.

Different results have been reported regarding the correlation between serum and BAL CTO activities and the radiological stages of sarcoidosis. In a previous study, a positive correlation was reported between all radiological stages and serum CTO activity (11). However, in further studies, serum CTO activity was reported to be significantly higher in stages III and IV than in stages 0-I. Additionally, it was higher in stage III than in stage 0. Conversely, it was found to be higher in a combined group of stages 0-II

compared to stages III-IV (17,21,23,32). In another study that included mostly patients with sarcoidosis stages I and II, there was a lack of correlation with the chest radiological stage. However, patients with an FVC or DLCO below normal values had significantly higher CTO activity (33). BAL CTO activity was shown to be correlated with quantitative HRCT score of lung volume affected by sarcoidosis and increased in stage II-III sarcoidosis (10). In another study, BAL CTO activity was associated with FVC and chest radiography scores higher in III-IV vs 0-II (30). In our study, no patients presented with stage III or IV disease. Although serum and BAL CTO activities were higher in stage II patients compared to stage I patients, this difference was not statistically significant.

In this study, there was no significant difference in CTO activity between patients with or without extrapulmonary sarcoidosis, consistent with some previous data (21,22). However, in some studies that included chronic sarcoidosis cases, patients with extrapulmonary involvement had significantly higher CTO activity than those with limited pulmonary disease, particularly in patients with abdominal organ involvement (34,35).

Calcium metabolism alterations are not rare in sarcoidosis. In our study group, two patients (4.8%) had hypercalciuria and one patient (2.4%) had hypercalcemia. We observed an inverse correlation between serum CTO activity and serum calcium levels (p= 0.026, r= -0.352). In a recent study, urinary calcium was reported to be correlated with serum CTO activity (34). Further studies are warranted to determine the relationship between CTO activity and calcium metabolism alterations.

Sarcoidosis is characterized by T-lymphocyte activation and accumulation of CD4-positive T-lymphocytes in the organs involved. BALF examination is a valuable tool in diagnosing sarcoidosis, as many patients exhibit elevated lymphocytosis and an increased CD4/CD8 ratio in BALF (2,36,37). Our study, the first to compare BAL CD4/CD8 ratio and serum CTO activity, found no significant difference between them. Thus, serum CTO activity proves to be as accurate as BAL CD4/ CD8 in the differential diagnosis of sarcoidosis.

Serum CTO activity was higher in patients with active sarcoidosis compared to those with inactive disease, suggesting its utility as a valuable marker for disease

monitoring (11,21,32). In a follow-up study of 95 newly diagnosed sarcoidosis cases, it was observed that patients who experienced relapses during follow- up had notably higher serum CTO activity at presentation compared to those who did not relapse. However, the CTO activity at diagnosis was not deemed sensitive or specific for predicting future relapses (33). In this study, we evaluated whether the CTO activity at diagnosis could predict progression during follow-up. Even though serum CTO activity was elevated in sarcoidosis patients requiring systemic therapy at diagnosis, no correlation was established between the serum or BAL CTO activity at diagnosis and the progression of the disease during follow-up. Additional studies are required to ascertain whether CTO activity at diagnosis can serve as an indicator of prognosis.

One limitation of this study was the absence of a control group comprising healthy individuals. This limitation arose due to ethical constraints regarding performing BAL on healthy subjects. The second limitation was the inability to conduct subgroup comparisons due to the small sample sizes of patients with various diseases within the non-sarcoidosis patient group. The third limitation arose from the absence of sarcoidosis patients classified under stages III and IV. Consequently, serum and BAL CTO levels could not be acquired from patients within these radiological stages, preventing comparisons among different radiological stages. Another limitation was that eleven patients with sarcoidosis were lost to follow-up after diagnosis, which hindered the evaluation of the prognosis for these individuals.

## CONCLUSION

In conclusion, this study represents the first instance in the literature where the diagnostic performance of serum CTO activity was found to be comparable to the BAL CD4/CD8 ratio. Furthermore, it has been demonstrated that serum CTO activity can be useful in the differential diagnosis of sarcoidosis. Although serum and BAL CTO activities were correlated with each other, and BAL CTO activity tended to be higher in patients with sarcoidosis, the role of BAL CTO activity in the differential diagnosis of sarcoidosis could not be demonstrated. Further studies with larger patient populations are needed to make more detailed comparisons with the diseases included in differential diagnosis. While serum CTO activity at

diagnosis does not predict progressive disease, it is correlated with the requirement for systemic therapy at diagnosis. Serial monitoring of CTO activity might prove valuable in monitoring disease progression during follow-up.

**Ethical Committee Approval:** This study was approved by the Ankara University Faculty of Medicine Clinical Research Ethics Committee (Decision no: 149-4614, Date: 06.04.2009).

### CONFLICT of INTEREST

The authors declare that they have no conflict of interest.

## AUTHORSHIP CONTRIBUTIONS

Concept/Design: GKB, GÇ Analysis/Interpretation: All of authors Data acqusition: All of authors Writing: All of authors

Clinical Revision: All of authors Final Approval: All of authors

## REFERENCES

1. * Statement on sarcoidosis. Joint Statement of the American Thoracic Society (ATS), the European Respiratory Society (ERS) and the World Association of Sarcoidosis and Other Granulomatous Disorders (WASOG) adopted by the ATS Board of Directors and by the ERS Executive Committee, February 1999. Am J Respir Crit Care Med 1999; 160(2): 736-55. *
2. * Baughman RP, Lower EE, du Bois RM. Sarcoidosis. Lancet 2003; 361(9363): 1111-8. https://doi.org/10.1016/ S0140-6736(03)12888-7*
3. * Crouser ED, Maier LA, Wilson KC, Bonham CA, Morgenthau AS, Patterson KC, et al. Diagnosis and detec- tion of sarcoidosis. An Official American Thoracic Society Clinical Practice Guideline. Am J Respir Crit Care Med 2020; 201(8): e26-e51. https://doi.org/10.1164/ rccm.202002-0251ST*
4. * Judson MA, Baughman RP, Thompson BW, Teirstein AS, Terrin ML, Rossman MD, et al. Two year prognosis of sar- coidosis: The ACCESS experience. Sarcoidosis Vasc Diffuse Lung Dis 2003; 20(3): 204-11.
  *
5. * Kurt I. Chitotriosidase: A new macrophage/phagocyte activation marker. J LSD 2011; 3(1): 8-15. *
6. * Renkema GH, Boot RG, Muijsers AO, Donker-Koopman WE, Aerts JM. Purification and characterization of human chitotriosidase, a novel member of the chitinase family of proteins. J Biol Chem 1995; 270(5): 2198-202. https:// doi.org/10.1074/jbc.270.5.2198*
7. * Van Eijk M, Van Roomen CP, Renkema GH, Bussink AP, Andrews L, Blommaart EF, et al. Characterization of human phagocyte-derived chitotriosidase, a component of innate immunity. Int Immunol 2005; 17(11): 1505-12. https://doi.org/10.1093/intimm/dxh328*
8. * Wajner A, Michelin K, Burin MG, Pires RF, Pereira ML, Giugliani R, et al. Biochemical characterization of chitotri- osidase enzyme: Comparison between normal individuals and patients with Gaucher and with Niemann-Pick diseas- es. Clin Biochem 2004; 37(10): 893-7. https://doi. org/10.1016/j.clinbiochem.2004.06.008*
9. * Malaguarnera L. Chitotriosidase: The yin and yang. Cell Mol Life Sci 2006; 63(24): 3018-29. https://doi. org/10.1007/s00018-006-6269-2*
10. * Bargagli E, Margollicci M, Perrone A, Luddi A, Perari MG, Bianchi N, et al. Chitotriosidase analysis in bronchoalveo- lar lavage of patients with sarcoidosis. Sarcoidosis Vasc Diffuse Lung Dis 2007; 24(1): 59-64. *
11. * Grosso S, Margollicci MA, Bargagli E, Buccoliero QR, Perrone A, Galimberti D, et al. Serum levels of chitotriosi- dase as a marker of disease activity and clinical stage in sarcoidosis. Scand J Clin Lab Invest 2004; 64(1): 57-62. https://doi.org/10.1080/00365510410004092*
12. * Guo Y, He W, Boer AM, Wevers RA, de Bruijn AM, Groener JE, et al. Elevated plasma chitotriosidase activity in various lysosomal storage disorders. J Inherit Metab Dis 1995; 18(6): 717-22. https://doi.org/10.1007/BF02436762*
13. * Lowry OH, Rosebrough NJ, Farr AL, Randall RJ. Protein measurement with the Folin phenol reagent. J Biol Chem 1951; 193(1): 265-75. https://doi.org/10.1016/S0021-*

*9258(19)52451-6*

14. * Scadding JG. Prognosis of intrathoracic sarcoidosis in England. A review of 136 cases after five years’ observa- tion. Br Med J 1961; 2(5261): 1165-72. https://doi. org/10.1136/bmj.2.5261.1165*
15. * Spagnolo P, Giulio R, Trisolini R, Sverzellati N, Baughman RP, Wells AU. Pulmonary sarcoidosis. Lancet Respir Med 2018; 6(5): 389-402. https://doi.org/10.1016/S2213-*

*2600(18)30064-X*

16. * Thillai M, Atkins CP, Crawshaw A, Hart SP, Ho LP, Kouranos V, et al. BTS clinical statement on pulmonary sarcoidosis. Thorax 2020; 0: 1-17. https://doi.org/10.1136/thorax- jnl-2019-214348*
17. * Bargagli E, Bianchi N, Margollicci M, Olivieri C, Luddi A, Coviello G, et al. Chitotriosidase and soluble IL-2 receptor: Comparison of two markers of sarcoidosis severity. Scand J Clin Lab Invest 2008; 68(6): 479-83. https://doi. org/10.1080/00365510701854975*
18. * Bargagli E, Margollicci M, Luddi A, Nikiforakis N, Perari MG, Grosso S, et al. Chitotriosidase activity in patients with interstitial lung diseases. Respir Med 2007; 101(10): 2176-81. https://doi.org/10.1016/j.rmed.2007.05.008*
19. * Bargagli E, Margollicci M, Nikiforakis N, Luddi A, Perrone A, Grosso S, et al. Chitotriosidase activity in the serum of patients with sarcoidosis and pulmonary tuberculosis. Respiration 2007; 74(5): 548-52. https://doi. org/10.1159/000100555*
20. * Brunner J, Scholl-Burgi S, Zimmerhackl LB. Chitotriosidase as a marker of disease activity in sarcoidosis. Rheumatol Int 2007; 27(12): 1171-2. https://doi.org/10.1007/ s00296-007-0363-0*
21. * Popevic S, Sumarac Z, Jovanovic D, Babic D, Stjepanovic M, Jovicic S, et al. Verifying sarcoidosis activity: Chitotriosidase versus ACE in sarcoidosis-a case-control study. J Med Biochem 2016; 35(4): 390-400. https://doi. org/10.1515/jomb-2016-0017*
22. * Tercelj M, Salobir B, Simcic S, Wraber B, Zupancic M, Rylander R. Chitotriosidase activity in sarcoidosis and some other pulmonary diseases. Scand J Clin Lab Invest 2009; 69(5): 575-8. https://doi. org/10.1080/00365510902829362*
23. * Bargagli E, Bennett D, Maggiorelli C, Di Sipio P, Margollicci M, Bianchi N, et al. Human chitotriosidase: A sensitive biomarker of sarcoidosis. J Clin Immunol 2013; 33(1): 264-70. https://doi.org/10.1007/s10875-012-9754-4*
24. * Boot RG, Hollak CE, Verhoek M, Alberts C, Jonkers RE, Aerts JM. Plasma chitotriosidase and CCL18 as surrogate markers for granulomatous macrophages in sarcoidosis. Clin Chim Acta 2010; 411(1-2): 31-6. https://doi. org/10.1016/j.cca.2009.09.034*
25. * Abaslı D. Measurement of plasma chitotriosidase and clinical applications (thesis). Ankara: Gülhane School of Medicine; 2008. *
26. * Kurt I, Abasli D, Cihan M, Serdar MA, Olgun A, Saruhan E, et al. Chitotriosidase levels in healthy elderly subjects. Ann N Y Acad Sci 2007; 1100: 185-8. https://doi.org/10.1196/ annals.1395.017*
27. * Csongradi A, Altorjay IT, Fulop GA, Enyedi A, Enyedi EE, Hajnal P, et al. Chitotriosidase gene polymorphisms and mutations limit the determination of chitotriosidase expression in sarcoidosis. Clin Chim Acta 2021; 513: 50-6. https://doi.org/10.1016/j.cca.2020.11.025*
28. * Enyedi A, Csongradi A, Altorjay IT, Beke GL, Varadi C, Enyedi EE, et al. Combined application of angiotensin converting enzyme and chitotriosidase analysis improves the laboratory diagnosis of sarcoidosis. Clin Chim Acta 2020; 500: 155-62. https://doi.org/10.1016/j. cca.2019.10.010*
29. * Kurt I, Olgun A, Hasimi A, Ozturk K, Erbil MK. Incidence of the 24-bp duplication in the chitotriosidase gene in Turkish population (452-P). 2007. *
30. * Harlander M, Salobir B, Zupancic M, Tercelj M. Bronchoalveolar lavage chitotriosidase activity as a bio- marker of sarcoidosis. Sarcoidosis Vasc Diffuse Lung Dis 2016; 32(4): 313-7. *
31. * d’Alessandro M, Carleo A, Cameli P, Bergantini L, Perrone A, Vietri L, et al. BAL biomarkers’ panel for differential diagnosis of interstitial lung diseases. Clin Exp Med 2020; 20(2): 207-16. https://doi.org/10.1007/s10238-020-*

*00608-5*

32. * Lopes MC, Amadeu TP, Ribeiro-Alves M, da Costa CH, Rodrigues LS, Bessa EJC, et al. Identification of active sar- coidosis using chitotriosidase and angiotensin-converting enzyme. Lung 2019; 197(3): 295-302. https://doi. org/10.1007/s00408-019-00219-2*
33. * Harlander M, Salobir B, Zupancic M, Dolensek M, Bavcar Vodovnik T, Tercelj M. Serial chitotriosidase measurements in sarcoidosis-two to five year follow-up study. Respir Med 2014; 108(5): 775-82. https://doi.org/10.1016/j. rmed.2014.02.002*
34. * Cameli P, Gonnelli S, Bargagli E, d’Alessandro M, Bergantini L, Favetta V, et al. The role of urinary calcium and chitotri- osidase in a cohort of chronic sarcoidosis patients. Respiration 2020; 99(3): 207-12. https://doi. org/10.1159/000505653*
35. * Bergantini L, Bianchi F, Cameli P, Mazzei MA, Fui A, Sestini P, et al. Prognostic biomarkers of sarcoidosis: A compara- tive study of serum chitotriosidase, ACE, lysozyme, and KL-6. Dis Markers 2019; 2019: 8565423. https://doi. org/10.1155/2019/8565423*
36. * Costabel U. CD4/CD8 ratios in bronchoalveolar lavage fluid: Of value for diagnosing sarcoidosis? Eur Respir J 1997; 10(12): 2699-700. https://doi. org/10.1183/09031936.97.10122699*
37. * Shen Y, Pang C, Wu Y, Li D, Wan C, Liao Z, et al. Diagnostic performance of bronchoalveolar lavage fluid CD4/CD8 ratio for sarcoidosis: A meta-analysis. EBioMedicine 2016; 8: 302-8. https://doi.org/10.1016/j.ebiom.2016.04.024*

